# Pathogenesis and prognosis of primary oral squamous cell carcinoma based on microRNAs target genes: a systems biology approach

**DOI:** 10.5808/gi.22038

**Published:** 2022-09-30

**Authors:** Amir Taherkhani, Shahab Shahmoradi Dehto, Shokoofeh Jamshidi, Setareh Shojaei

**Affiliations:** 1 Research Center for Molecular Medicine, Hamadan University of Medical Sciences, Hamadan, Iran; 2 Department of Oral and Maxillofacial Pathology, Faculty of Dentistry, Hamadan University of Medical Sciences, Hamadan, Iran; 3 Dental Research Center, Department of Oral and Maxillofacial Pathology, School of Dentistry, Hamadan University of Medical Sciences, Hamadan, Iran

**Keywords:** biomarkers, cancer, head and neck, oral, pathogenesis, prognosis

## Abstract

Oral squamous cell carcinoma (OSCC) is the most prevalent head and neck malignancy, with frequent cervical lymph-node metastasis, leading to a poor prognosis in OSCC patients. The present study aimed to identify potential markers, including microRNAs (miRNAs) and genes, significantly involved in the etiology of early-stage OSCC. Additionally, the main OSCC's dysregulated Gene Ontology annotations and significant signaling pathways were identified. The dataset GSE45238 underwent multivariate statistical analysis in order to distinguish primary OSCC tissues from healthy oral epithelium. Differentially expressed miRNAs (DEMs) with the criteria of p-value < 0.001 and |Log2 fold change| > 1.585 were identified in the two groups, and subsequently, validated targets of DEMs were identified. A protein interaction map was constructed, hub genes were identified, significant modules within the network were illustrated, and significant pathways and biological processes associated with the clusters were demonstrated. Using the GEPI2 database, the hub genes' predictive function was assessed. Compared to the healthy controls, main OSCC had a total of 23 DEMs. In patients with head and neck squamous cell carcinoma (HNSCC), upregulation of *CALM1*, *CYCS*, *THBS1*, *MYC*, *GATA6*, and *SPRED3* was strongly associated with a poor prognosis. In HNSCC patients, overexpression of *PIK3R3*, *GIGYF1*, and *BCL2L11* was substantially correlated with a good prognosis. Besides, “proteoglycans in cancer” was the most significant pathway enriched in the primary OSCC. The present study results revealed more possible mechanisms mediating primary OSCC and may be useful in the prognosis of the patients with early-stage OSCC.

## Introduction

Oral cancer (OC) is a global public health problem, accounting for the fourth highest frequency of malignancy [1]. Oral squamous cell carcinoma (OSCC) is the most common histological type of OC [2-4]. The progression of OSCC is step-by-step and is characterized by the transformation of normal oral epithelium to precancerous lesions and cancerous squamous epithelial tissues [5]. Human papillomavirus infections, smoking, consuming alcohol, and having poor oral hygiene are a few risk factors that have been linked to OSCC [6]. The major treatment approach for individuals with late stages of OSCC is surgery, which may be combined with chemotherapy and radiation. This is because many OSCC patients are often diagnosed in advanced stages. OSCC has remained one of the mortal malignancies worldwide, with a 5-year survival rate of approximately 50%, indicating that the prognosis of OSCC patients is poor [7-12]. Thus, illustrating the exact mechanisms and markers underlying the development of OSCC may elevate patient survival [8,10].

Protein-coding genes include approximately 2% of the entire human genome, and therefore, the regulatory role of non-coding RNAs (ncRNAs) in signaling pathways, biological processes (BPs), and networks is inevitable [13,14]. The crucial involvement of ncRNAs (practically microRNAs [miRNAs]) as tumor suppressors or tumor promoter genes in many kinds of malignancies, including OC, has been supported by a sizable number of research published in the last 10 years [15,16]. A group of ncRNAs with a length of 18–22 nucleotides are known as miRNAs. These small RNAs either encourage mRNA degradation or restrict mRNA translation to adversely affect the expression of their target gene(s) [15]. It was reported that miRNAs control up to 50% of the human gene expression [17]. The miRNAs involve in many critical biological procedures, including cell development, metabolism, proliferation, and signal transduction [18].

The present study hypothesized that the dysregulation of miRNAs in the early-stage OSCC tumor specimens (stages I and II) compared to their adjacent normal epithelium leads to the aberrant expression of their target genes. Therefore, differentially expressed miRNAs (DEMs) in early-stage OSCC tumor tissues compared with the adjacent healthy controls were identified using the multivariate statistical analysis (p-value < 0.001; |Log2 fold change (FC)| > 1.585), and the differential power of DEMs was evaluated using the support vector machine (SVM) algorithm. Additionally, it was hypothesized that the DEMs-targets, which are crucial in the pathogenesis of early-stage OSCC, might serve as prognostic indicators for the condition. In order to study the prognostic significance of the hub genes inside the network, we created a protein interaction map (PIM) based on the DEMs-targets. Furthermore, based on DEMs-targets and important clusters in the PIM, Gene Ontology (GO) annotation and pathway enrichment studies were carried out. The aims of the present study were followed by re-analyzing the dataset GSE45238, which was established by Shiah et al. [19] to monitor miRNA expression profile in OSCC tissue specimens and their adjacent non-cancerous epithelium. All tissue samples were collected from patients who underwent curative surgery from 1999 to 2010 at the National Cheng Kung University Hospital (Tainan, Taiwan). Liquid nitrogen was used to store the freshly frozen samples until usage. The Institutional Human Experiment and Ethics Committee accepted the trial, and tumor staging was carried out using the American Joint Committee on Cancer staging method (HR-97-100). The present results may be beneficial to reach a more pleasant prognosis in the patients with early-stage OSCC, leading to appropriate treatments on behalf of patients. The present study was confirmed by the Ethics Committee of Hamadan University of Medical Sciences, Hamadan, Iran (ethics no. IR.UMSHA.REC.1400.722).

## Methods

### MiRNA expression dataset recovery

The miRNA expression dataset GSE45238 [19] was attained from the National Center for Biotechnology Information Gene Expression Omnibus, available from: http://www.ncbi.nlm.nih.gov/geo [20]. The GSE45238 contained 80 samples including stage I OSCC (n = 1), stage II OSCC (n = 14), stage III OSCC (n = 10), and stage IV OSCC (n = 15), and their adjacent non-tumor epithelium, based on the GPL8179 platform (Illumina Human v2 MicroRNA expression bead chip, Illumina, San Diego, CA, USA). To illustrate markers involved in primary OSCC, a new dataset was selected from the GSE45238 for further analyses, including 15 early-stage OSCC tissue samples (stage I [n = 1] and stage II [n = 14]) and their corresponding healthy control specimens.

### Statistical analysis

The normalized dataset after preprocessing had 30 observations (15 early-stage OSCC and 15 nearby healthy controls) and 858 characteristics (miRNA IDs). Using the "ropls," "limma," and "genefilter" packages from the R language (version 4.2.0) [21], the advanced supervised orthogonal partial least squares discriminant analysis (OPLS-DA) was used to find DEMs in early-stage OSCC samples as opposed to healthy controls. DEMs with the criteria of p-value < 0.001 and 1/3 > FC > 3 (absolute Log2 FC [|Log2 FC|] > 1.585) were considered significant.

### SVM classifier

To evaluate the differential power of DEMs in early-stage OSCC tissues compared to the healthy controls, the SVM algorithm with the linear kernel was constructed using the e1071 package (version 1.6-8) within the R environment (https://cran.rproject.org/web/packages/e1071). The model's sensitivity, specificity, and accuracy were calculated using the leave-one-out (LOO) approach, and cross-validation was carried out by repeating LOO 100 times [22]. Furthermore, the receiver operating characteristic (ROC) curve was drawn, and the area under the curve (AUC) value was then calculated to appraise the model's predictive power.

### PIM, module, signaling pathway, and GO annotation analyses

MiRWalk 2.0 database [23] was utilized to illustrate the experimentally validated targets of DEMs. In this regard, only targets included in the miRTarBase [24] with a score of 1 were assigned as validated DEMs-targets. Then, all validated targets were used to illustrate cellular components (CCs) and molecular functions (MFs) affected in the early-stage OSCC. The STRING knowledge base webserver (HTTP://string-db.org) [25] was used to unravel possible connections among the target gene. Subsequently, the Cytoscape (version 3.9.1) [26] was utilized to build a PIM from the targets. The MCODE plugin was used to show major linked areas within the network after single genes were removed from the PIM [27]. Only modules with the following criteria were assigned important and considered for further pathway enrichment and BP annotation analysis: minimum MCODE score = 3, the minimum number of genes = 10, Max depth = 100, Degree cutoff = 2, k-score = 2, and node score cutoff = 0.2. The Pathway and GO annotation enrichment analyses were executed using the g:Profiler tool (https://biit.cs.ut.ee/gprofiler/gost) [28], and the terms with the criteria of false discovery rate (FDR) < 0.05 and number of enriched genes ≥ 10 were considered significant. The Network Analyzer tool was used to determine the centrality parameters of the network's nodes in addition to topological analysis, and hub status was given to the genes whose degree and betweenness centrality values were higher than the mean of the network's vertexes.

### Survival and boxplot analyses

The prognostic impact of the hub genes associated with the early-stage OSCC was studied utilizing Kaplan-Meier curves. The survival analysis was conducted using GEPIA2 database (http://gepia2.cancer-pku.cn/#survival) [29]. Re-analyzing RNA sequencing resources, such as The Cancer Genome Atlas [30] and Genotype-Tissue Expression [31] databases (which are linked to human cancer diseases), is made possible by GEPIA2. This process yields more accurate results for survival, box plot, and correlation analysis, as well as similar gene identification in cancer patients as compared to healthy controls. Cox proportional hazards regression model calculated the corrected hazard ratios (HR) and 95% confidence intervals of the hub genes. Kaplan-Meier curves with the log-rank test p-value and HR p-value as < 0.05 were assigned significant. Moreover, the expression patterns of prognostic markers in head and neck squamous cell carcinoma (HNSCC) tissue samples and healthy controls were studied using the valuable data from the GEPI2A database.

## Results

### Identification of DEMs and their targets in early-stage OSCC

**Fig. 1.**
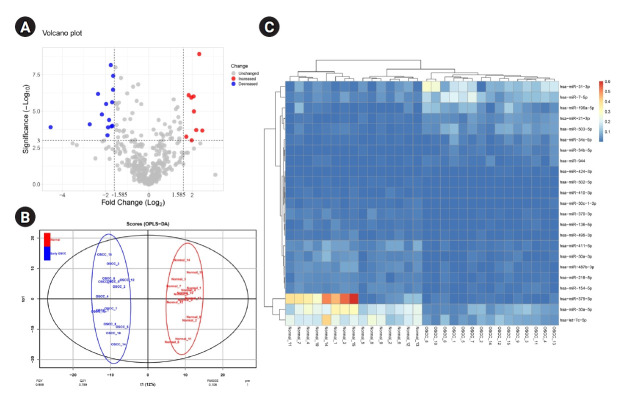
(A) The volcano plot of miRNAs in the early-stage OSCC tissues compared to the normal epithelium. (B) Score plot in the predictive (x-axis) and orthogonal (y-axis) components of dataset GSE45238 achieved from the OPLS-DA model. (C) Heat-map of DEMs in early-stage OSCC tissues compared with the healthy oral epithelium. OSCC, oral squamous cell carcinoma; OPLS-DA, orthogonal partial least squares discriminant analysis; DEM, differentially expressed miRNA.

OPLS-DA was performed to differentiate early-stage OSCC tissue samples (n = 15) from healthy controls (n = 15). The model strongly classified two datasets with the following parameters: R^2^X = 0.223, R^2^Y = 0.959, and Q^2^ = 0.789. At a p-value of 0.001 and |Log2 FC >| 1.585, 23 DEMs, including nine up-regulated and 14 down-regulated miRNAs, were identified in the early-stage OSCC tissues compared with non-cancerous specimens (Table 1). Six hundred seventy-two distinct genes were shown in the MiRWalk 2.0 database as verified targets for DEMs. The dataset GSE45238's volcano plot is shown in Fig. 1A thanks to the Shiny applications tool (available at https://huygens.science.uva.nl/) [32]. Fig. 1B displays the OPLS-DA scores plot. Using an unsupervised hierarchical clustering approach in the R environment (version 4.2.0), all early-stage OSCC samples were effectively separated from their nearby healthy controls (Fig. 1C).

### SVM modeling and cross-validation

**Fig. 2.**
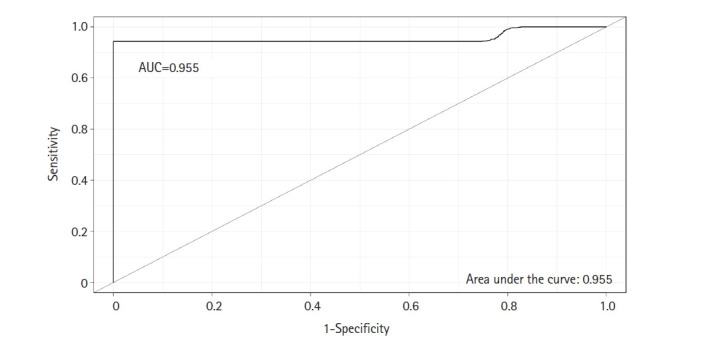
ROC curve was completed to test the predictive power of the SVM classifier with a linear kernel. The AUC was calculated as 0.955. ROC, receiver operating characteristic; SVM, support vector machine; AUC, area under the curve.

A classifier model was constructed using the SVM algorithm via linear kernel to evaluate the differential power of DEMs in early-stage OSCC samples compared to the normal oral epithelium. The dataset included 23 DEMs and 30 observations (early-stage OSCC = 15; normal = 15). The SVM model has the following parameters: cost = 0.01, number of support vectors = 16, and SVM-type = C-classification. The average value of sensitivity, specificity, and accuracy for the SVM was estimated as 100%, 95%, and 97%, respectively, after completing 100 rounds of LOO cross-validations. The ROC curve of model was also achieved using the “Epi” package in R language (Fig. 2), in which the AUC of the SVM was determined as 0.955. Supplementary Table 1 shows the details of statistics of the model over the 100 times cross-validations.

### PIM network, clustering, and functional analyses

**Fig. 3.**
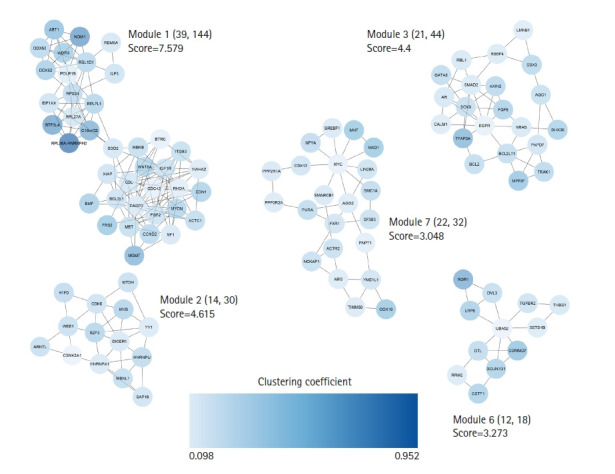
Clustering analysis was performed with the MCODE plugin. Five salient modules were detected in the PIM linked to the early-stage OSCC. PIM, protein interaction map; OSCC, oral squamous cell carcinoma.

**Fig. 4.**
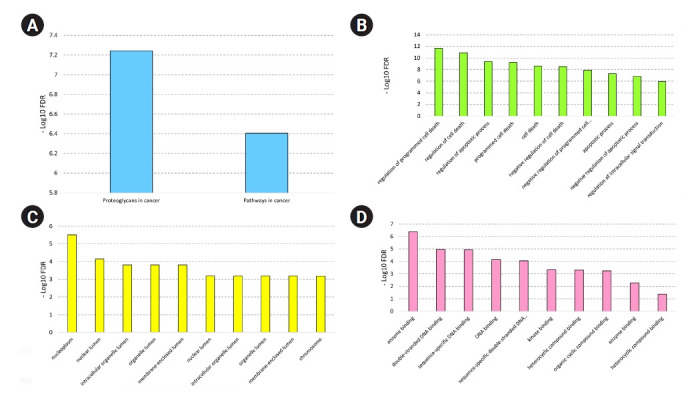
Most significant signaling pathways (A), biological processes (B), cellular components (C), and molecular functions (D) are affected in the early-stage OSCC. The X-axis shows the name of the term. Y-axis demonstrates the minus value of the Log10 FDR. FDR, false discovery rate; OSCC, oral squamous cell carcinoma.

A total of 672 unique targets were uploaded into the STRING database, and a primary protein-protein interaction (PPI) network was constructed with a confidence score of ≥ 0.4. A PIM meeting the criterion of 605 proteins and 2,322 interactions was created after unconnected nodes were removed, and it was then imported into the Cytoscape program for further analysis. The MCODE program found five major modules in all, including clusters Nos. 1, 2, 3, 6, and 7 (Fig. 3). Besides, 85 genes indicated a higher degree and betweenness centralities compared to the average value of the mentioned parameters within the PPI network therefore, were assigned as hub genes associated with the pathogenesis of early-stage OSCC (Supplementary Table 2). The average value for degree and betweenness centrality was determined as 7.68 and 0.0060, respectively. At an FDR of 0.05 and a number of enriched genes ≥ 10, a total of 11 MFs, 26 CCs, 174 BPs, and two pathways were found to be significantly dysregulated in early-stage OSCC. Fig. 4 shows top-10 significant MFs, CCs, and BPs, as well as two signaling pathways enriched in the early-stage of OSCC.

### Prognostic role of the hub genes and expression analysis

**Fig. 5.**
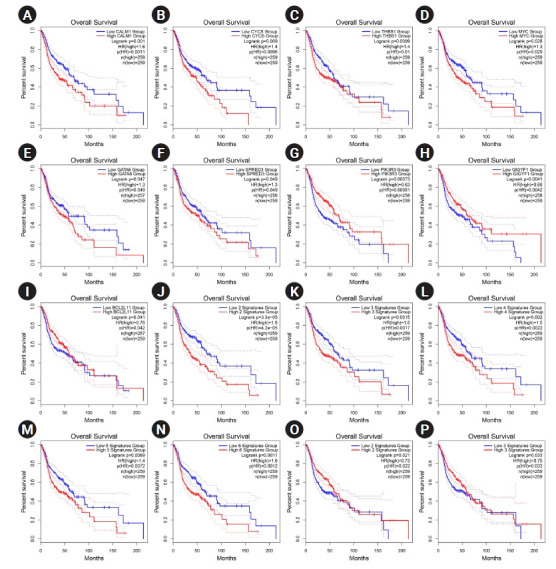
(A‒P) Prognostic impact of *CALM1* (A), *CYCS* (B), *THBS1* (C), *MYC* (D), *GATA6* (E), *SPRED3* (F), *PIK3R3* (G), *GIGYF1* (H), and *BCL2L11* (I) was significant in HNSCC. Prognostic role of the panel of genes is also exhibited. The X-axis and Y-axis represent the survival time of patients with HNSCC and the survival probability, respectively. The dotted lines are 0.95 confidence intervals. HNSCC, head and neck squamous cell carcinoma; HR, hazard ratio.

The overexpression of six hub genes, including *CALM1*, *CYCS*, *THBS1*, *MYC*, *GATA6*, and *SPRED3*, was strongly related with a poor prognosis in patients with HNSCC, according to the survival analysis performed using the GEPIA2 database. In contrast, upregulation of three of hubs, including *PIK3R3*, *GIGYF1*, and *BCL2L11* was considerably related to a favorable prognosis in HNSCC patients (log-rank test and HR p-values < 0.05). Thus, a prognostic panel including *CALM1* and *CYCS* revealed the most significant result with the HR = 1.8 and p (HR) = 0.000042 (Table 2, Fig. 5).

**Fig. 6.**
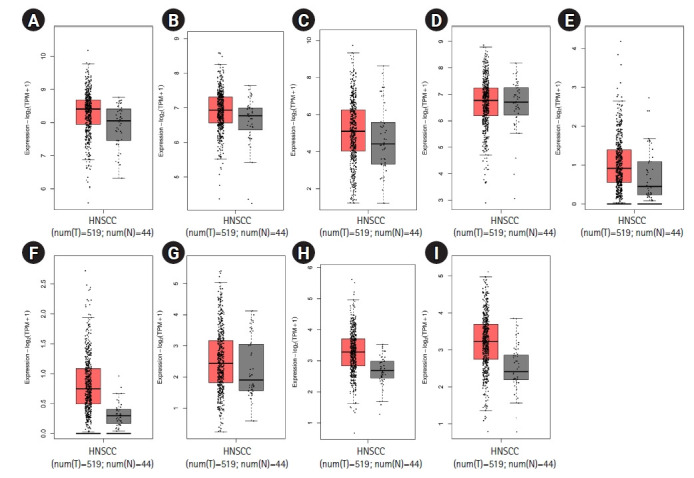
Gene expression of prognostic markers in HNSCC from the expression analysis in the GEPIA2 database. Box plots are based on 44 normal tissues (gray color) and 519 HNSCC samples (red color). The boxplot analysis show overexpression of *CALM1* (A), *CYCS* (B), *THBS1* (C), *MYC* (D), *GATA6* (E), *SPRED3* (F), *PIK3R3* (G), *GIGYF1* (H), and *BCL2L11* (I) in HNSCC. However, *MYC* shows a mild upregulation in HNSCC. HNSCC, head and neck squamous cell carcinoma.

According to the expression analysis from GEPIA2, all prognostic markers in HNSCC exhibited higher expression in cancerous tissues than the healthy controls (Fig. 6).

## Discussion

A dismal prognosis is associated with OC, which continues to be one of the most prevalent carcinomas globally. Additionally, the paucity of reliable markers to forecast OC development contributes to treatment failures [33,34]. The current investigation discovered 23 miRNAs that were differently expressed between healthy control epithelium and primary OSCC tissues. The miR-21-3P indicated the most overexpression in primary OSCC tissues compared to the adjacent normal specimens with the criteria of Log2 FC = 2.33 and p-value of 1.24E-09. Previous studies have assigned miR-21 as a predictive, diagnostic, and prognostic marker in several human cancers [35]. MiR-21 overexpression is linked to increased cellular proliferation, aggressive behavior, and metastasis because miR-21 targets multiple tumor suppressor genes [36-39]. According to Amirfallah et al. [40], overexpression of miR-21-3p was substantially linked to advanced-stage breast cancer, lymph-node metastases, a low likelihood of survival, and big tumor sizes. The authors reported that miR-21-3p overexpression was related to the downregulation of tumor suppressor genes, suggesting miR-21-3p as a potential biomarker in breast tumors. In addition, Gao et al. [41] showed that miR-21-3p upregulation was significantly associated with stemness maintenance of cancer stem cells and a high risk of esophageal squamous cell carcinoma.

Moreover, the present study indicated that has-miR-375-3p was the most under-expressed miRNA in primary OSCC than normal adjacent tissues (Log2 FC = ‒4.54 and p = 1.24E-04). Crimi et al. [42] performed a study to determine the expression levels of four miRNAs including hsa-miR-375-3p, hsa-miR-133a-3p, hsa-miR-503-5p, and hsa-miR-196a-5p in liquid biopsies obtained from OC patients and healthy individuals. With p-values of 0.0001 and 0.001, respectively, the droplet digital polymerase chain reaction revealed lower plasma levels of hsa-miR-133a-3p and hsa-miR-375-3p in OC compared to healthy controls. Additionally, Crimi et al. [42] demonstrated that hsa-miR-375-3p and hsa-miR-133a-3p were highly sensitive and specific diagnostic indicators for OC, with AUCs of 0.96 and 0.86, respectively. The significant role of hsa-miR-375-3p in the development of colorectal cancer (CRC) was shown. Xu et al. [43] reported a significant association between miR-375-3p downregulation and a dismal survival rate in CRC patients. Besides, upregulation of miR-375-3p elevated the sensitivity of CRC cells to 5-fluorouracil‒based chemotherapy through negative regulation of YAP1 and SP1.

Overexpression of the DEMs target genes *CALM1*, *CYCS*, *THBS1*, *MYC*, *GATA6*, and *SPRED3* was strongly associated with a poor prognosis in patients with HNSCC. Additionally, patients with HNSCC who had upregulation of *PIK3R3*, *GIGYF1*, and *BCL2L11* had better prognoses. Calmodulin (CALM) is a major conserved calcium ion receptor protein in eukaryotic cells and is encoded by CALM1, CALM2, and CALM3 in humans [44]. The CALM/Ca^2+^ binds to the phosphoinositide 3-kinase α (PI3Kα), leading to the hyperactivation of PI3Kα/Akt/mammalian target of rapamycin signaling pathway, cell proliferation, differentiation, motility, and development [45-47]. The overexpression of CALM1 was indicated in many cancers including prostate cancer [48], bladder cancer [49], and nasopharyngeal carcinoma [50].

A significant part in promoting the signaling pathways connected to programmed cell death is played by cytochrome c (CYCS) [51]. However, CYCS is a poor prognostic indicator in the majority of malignancies, which may be a result of a malignant tissue response [52,53]. Thrombospondin-1 (THBS1) is an adhesive glycoprotein. THBS1 plays a role in wound healing by mediating cell adhesion, proliferation, and migration. Moreover, THBS1 is involved in the cancer cell and metastasis [54]. The upregulation of THBS1 was illustrated in different cancer cells such as ovarian cancer [55], mammary cancer [56], and thyroid cancer [57]. Hayashido et al. [54] studied the expression of THBS1 at the protein level in OSCC tissues using the immunohistochemical assay. In the OSCC cells, THBS1 induced the production of matrix metalloproteinase-9, which increased proteolytic activity and haptotactic migration, according to the findings of the research by Hayashido et al. [54]. Furthermore, Xiao et al. [9] showed that exosomes carrying the THBS1 protein that were released from OC tissues might facilitate the conversion of macrophages into tumor-associated macrophages that resemble M1.Myc proto-oncogene protein (MYC) is a well-known transforming gene in many cancers such as breast [58] and lung cancer [59], as well as hepatocellular carcinoma [60]. MYC is up-regulated in 80% of the OSCC cases [61] and has shown high frequency by Genome-wide profiling of OSCC [62]. Wang et al. [10] reported that the miR-1294 targeted the 3'-untranslated region of c-MYC and led to the downregulation of MYC mRNA in OSCC cancer cells.

GATA6 (GATA binding protein 6) is a transcription factor involved in a GATA family regulating several genes participating in tissue morphogenesis and cell fate decision making [63]. GATA6 has been described as an independent prognostic marker in ovarian cancer and plays a key role in the differentiation and invasion of tumor cells [64]. GATA6 upregulation has been shown in several tumors in the past, including CRC [65], gastric cancer [66], and cholangiocarcinoma [67]. According to Zhai and Luo [8], OSCC cell lines (HN4, HN6, SCC9, and Cal27) overexpress GATA6 in comparison to human normal oral mucosal epithelial keratinocytes. Furthermore, GATA6 knockdown suppressed the colony formation, proliferation, migration, and invasion of cancerous cells. According to a previous study, GATA6 expression was diminished by miRNA-506 and attenuated the progression of OSCC [68].

Sprouty-related, EVH1 domain-containing protein 3 (*SPRED3*) downregulates the Ras/Raf/mitogen-activated protein kinase (MAPK) signaling pathway during the organogenesis [69]. It was indicated that *SPRED3* is frequently loosed in glioblastoma [70]. Furthermore, He et al. [71] showed that non‒small cell lung cancer with repressed *SPRED3* gene expression increased Ras/Raf/MAPK signaling, resulting in resistance to epidermal growth factor receptor–tyrosine kinase inhibitors. According to the present results, the has-miR-370-3p was significantly down-regulated in the early-stage OSCC tissues than the healthy controls (p = 0.0000398; Log2 FC = ‒1.84), which may be hypothesized that SPRED3 is overexpressed in the early-stage OSCC tissues, which may be in line with the cancer response. However, this requires further confirmation.

In order to demonstrate important pathways and GO annotations impacted in main OSCC, gene set enrichment analysis was performed. The genes linked to prominent clusters in the PPI network connected to the etiology of the illness were used for this. The most important route, then, was "proteoglycans in cancer" (KEGG:05200). Proteoglycans are classified as highly glycosylated proteins involved in various parts of tissues, including the cell membrane, extracellular matrix, and pericellular space. These heavy molecules mediate many important BPs associated with angiogenesis, tissue regeneration, cell growth, and motility. Numerous forms of cancer have been linked to aberrant proteoglycan expression [72]. In solid tumors and hematological malignancies, proteoglycans make cancer cells more aggressive [73]. Additionally, proteoglycans participate in the receptor-ligand interaction between cancer cells and their environment, which may facilitate cell migration and increase the number of circulating tumor cells [74]. Besides, previous studies indicated that heparan sulfate proteoglycans (HSPGs) are involved in cellular differentiation and proliferation, apoptosis, immune evasion, angiogenesis, and matrix remodeling in cancer. Therefore, targeting proteoglycans and HSPGs could be assigned as a practical therapeutic approach in cancer [75,76].

In conclusion, the present study identified 23 DEMs in primary OSCC tissues compared to the normal oral epithelium. MiR-21-3p and miR-375-3p were the most salient up-regulated and down-regulated miRNAs in primary OSCC tissues compared to healthy controls, respectively. It has been hypothesized that DEMs contribute to OSCC's onset or undergo dysregulation in response to a tumor mass. Additionally, there was a strong correlation between a poor outcome and the upregulation of *CALM1*, *CYCS*, *THBS1*, *MYC*, *GATA6*, *SPRED3*, and the under-expression of *PIK3R3*, *GIGYF1*, and *BCL2L11* in HNSCC patients. Furthermore, “proteoglycans in cancer” was demonstrated as the most salient signaling pathway affected in primary OSCC. These results may help the prognosis of the patients with early-stage OSCC, leading to more useful therapeutic approaches.

## Figures and Tables

**Table 1.** A total of 23 miRNAs were identified to be differentially expressed in early OSCC tissues compared to healthy controls

miRNA name	Log2 FC	|Log2 FC|	p-value
hsa-miR-21-3p	2.33	2.33	1.24E-09
hsa-miR-503-5p	2.07	2.07	9.64E-07
hsa-miR-31-3p	2.08	2.08	1.03E-05
hsa-miR-196a-5p	2.19	2.19	1.95E-04
hsa-miR-34b-5p	2.47	2.47	2.11E-04
hsa-miR-34c-5p	1.98	1.98	9.73E-04
hsa-miR-424-3p	1.96	1.96	1.16E-06
hsa-miR-7-5p	1.84	1.84	8.04E-07
hsa-miR-944	1.73	1.73	5.53E-04
hsa-miR-411-5p	‒2.35	2.35	6.61E-07
hsa-miR-30a-3p	‒2.17	2.17	1.64E-05
hsa-miR-410-3p	‒2.73	2.73	7.88E-05
hsa-miR-375-3p	‒4.54	4.54	1.24E-04
hsa-miR-487b-3p	‒1.97	1.97	3.16E-06
hsa-miR-136-5p	‒1.91	1.91	4.41E-04
hsa-miR-495-3p	‒1.89	1.89	1.27E-04
hsa-miR-370-3p	‒1.84	1.84	3.98E-05
hsa-miR-30a-5p	‒1.76	1.76	7.06E-09
hsa-miR-502-5p	‒1.73	1.73	1.10E-04
hsa-miR-218-5p	‒1.70	1.70	2.45E-06
hsa-miR-30c-1-3p	‒1.68	1.68	1.00E-04
hsa-miR-154-5p	‒1.66	1.66	3.38E-07
hsa-let-7c-5p	‒1.65	1.65	3.82E-08

OSCC, oral tongue squamous cell carcinoma; FC, fold change.

**Table 2.** Nine of the hub genes in early OSCC significantly demonstrated a prognostic role in HNSCC

	HR (high)	p (log-rank test)	p (HR)
Gene symbol (gene label)			
Single-gene			
*CALM1* (A)	1.6	0.001	0.0011
*CYCS* (B)	1.4	0.009	0.0096
*THBS1* (C)	1.4	0.0099	0.01
*MYC* (D)	1.3	0.028	0.029
*GATA6* (E)	1.3	0.047	0.049
*SPRED3* (F)	1.3	0.049	0.049
*PIK3R3* (G)	0.63	0.00073	0.00081
*GIGYF1* (H)	0.68	0.0041	0.0042
*BCL2L11* (I)	0.76	0.041	0.042
Prognostic panel			
Combination of genes			
A + B	1.8	0.000033	0.000042
A + B + C	1.5	0.0015	0.0017
A + B + C + D	1.5	0.002	0.0022
A + B + C + D + E	1.4	0.0069	0.0072
A + B + C + D + E + F	1.6	0.0011	0.0012
G + H	0.73	0.021	0.022
G + H + I	0.75	0.033	0.033

OSCC, oral squamous cell carcinoma; HNSCC, head and neck squamous cell carcinoma; HR, hazard ratio.

## Supplementary Materials

Supplementary Table 1. The details of statistics of the SVM model over the 100 times cross-validations

Supplementary Table 2. A total of 85 hub genes associated with the etiology of early OSCC
