# Supplementary material for: Pathogenesis and prognosis of primary oral squamous cell carcinoma based on microRNAs target genes: a systems biology approach
Source: Genomics Inform. 2022 Sep 30;20(3):e27. doi: 10.5808/gi.22038 (PMC9576470; doi:10.5808/gi.22038)
Supplement: Supplementary Table 1. — The details of statistics of the SVM model over the 100 times cross-validations [file gi-22038suppl1.pdf]

Supplementary Table 1. The details of statistics of the SVM model over the 100 times cross-validations

No. of leave one out	Sensitivity	Specificity	Accuracy
1	1	1.00	1.00
2	1	0.91	0.97
3	1	1.00	1.00
4	1	0.82	0.93
5	1	0.93	0.97
6	1	1.00	1.00
7	1	1.00	1.00
8	1	1.00	1.00
9	1	1.00	1.00
10	1	0.88	0.93
11	1	1.00	1.00
12	1	0.89	0.93
13	1	0.94	0.97
14	1	1.00	1.00
15	1	0.93	0.97
16	1	1.00	1.00
17	1	1.00	1.00
18	1	1.00	1.00
19	1	1.00	1.00
20	1	0.67	0.87
21	1	1.00	1.00
22	1	1.00	1.00
23	1	0.79	0.90
24	1	1.00	1.00
25	1	0.94	0.97
26	1	0.94	0.97
27	1	1.00	1.00
28	1	0.93	0.97
29	1	0.85	0.93
30	1	0.93	0.97
31	1	1.00	1.00
32	1	0.93	0.97
33	1	1.00	1.00
34	1	0.94	0.97
35	1	0.91	0.97
36	1	1.00	1.00
37	1	1.00	1.00
38	1	0.94	0.97
39	1	1.00	1.00

40	1	0.93	0.97
41	1	0.94	0.97
42	1	1.00	1.00
43	1	1.00	1.00
44	1	0.93	0.97
45	1	0.93	0.97
46	1	0.95	0.97
47	1	0.76	0.87
48	1	1.00	1.00
49	1	0.80	0.87
50	1	1.00	1.00
51	1	0.87	0.93
52	1	0.94	0.97
53	1	0.89	0.97
54	1	1.00	1.00
55	1	1.00	1.00
56	1	0.92	0.97
57	1	0.75	0.87
58	1	1.00	1.00
59	1	0.92	0.97
60	1	1.00	1.00
61	1	0.93	0.97
62	1	1.00	1.00
63	1	0.81	0.90
64	1	0.93	0.97
65	1	1.00	1.00
66	1	0.95	0.97
67	1	0.91	0.97
68	1	0.93	0.97
69	1	0.89	0.93
70	1	1.00	1.00
71	1	0.94	0.97
72	1	1.00	1.00
73	1	1.00	1.00
74	1	0.92	0.97
75	1	1.00	1.00
76	1	0.95	0.97
77	1	1.00	1.00
78	1	0.85	0.90
79	1	1.00	1.00
80	1	1.00	1.00
81	1	0.93	0.97
82	1	0.94	0.97

83	1	1.00	1.00
84	1	0.90	0.97
85	1	1.00	1.00
86	1	1.00	1.00
87	1	0.93	0.97
88	1	0.94	0.97
89	1	1.00	1.00
90	1	1.00	1.00
91	1	0.93	0.97
92	1	0.82	0.93
93	1	0.87	0.93
94	1	1.00	1.00
95	1	0.92	0.97
96	1	1.00	1.00
97	1	1.00	1.00
98	1	0.94	0.97
99	1	1.00	1.00
100	1	1.00	1.00

SVM, support vector machine.
