# Supplementary material for: Pathogenesis and prognosis of primary oral squamous cell carcinoma based on microRNAs target genes: a systems biology approach
Source: Genomics Inform. 2022 Sep 30;20(3):e27. doi: 10.5808/gi.22038 (PMC9576470; doi:10.5808/gi.22038)
Supplement: Supplementary Table 2. — A total of 85 hub genes associated with the etiology of early OSCC [file gi-22038suppl2.pdf]

Supplementary Table 2. A total of 85 hub genes associated with the etiology of early OSCC

Gene ID	Degree	Betweenness
MYC	95	0.1355
EGFR	84	0.1449
CDC42	62	0.0814
UBA52	58	0.0810
RHOA	57	0.0521
CASP3	53	0.0305
SMAD2	40	0.0331
DICER1	39	0.0358
BTRC	39	0.0336
NRAS	39	0.0222
CYCS	37	0.0479
AGO2	37	0.0469
FGF2	36	0.0280
APP	35	0.0329
CBL	33	0.0330
YWHAZ	33	0.0267
IGF1R	33	0.0224
HNRNPA1	32	0.0188
BCL2L1	31	0.0182
AR	30	0.0180
CDK6	30	0.0088
TFRC	29	0.0383
NF1	29	0.0167
YY1	29	0.0104
SRSF2	27	0.0225
MET	27	0.0146
RBBP4	26	0.0123
SOX9	25	0.0151
BCL2L11	25	0.0098
AGO1	25	0.0068
CANX	24	0.0354
POLR1B	24	0.0203
SOD2	24	0.0164
PPP2R1A	24	0.0129
XIAP	24	0.0109
CSNK2A1	23	0.0240
ITGB3	23	0.0146
RBM8A	23	0.0116
LMNB1	22	0.0156
CUL3	21	0.0217

RPL27A	21	0.0172
CCNT1	21	0.0126
TGFBR2	21	0.0084
THBS1	21	0.0081
CALM1	20	0.0170
FXR1	20	0.0127
WEE1	20	0.0066
PPP2R2A	19	0.0097
POLR2D	18	0.0102
RBL1	18	0.0082
SREBF1	17	0.0110
ILF3	17	0.0094
PIK3R3	17	0.0077
SETD1B	17	0.0068
SUPT16H	17	0.0065
LIN28A	16	0.0093
EIF1AX	15	0.0138
MRPS16	15	0.0105
GRIN2A	15	0.0099
TGOLN2	15	0.0095
LDHA	15	0.0073
RDX	14	0.0118
RRM2	14	0.0086
SUOX	13	0.0108
SORT1	13	0.0102
TFAM	13	0.0074
TPI1	13	0.0073
NUP43	13	0.0068
GGA3	12	0.0073
GDNF	12	0.0072
SPATA5	11	0.0179
SNX9	11	0.0103
GATA6	11	0.0068
CASK	10	0.0091
PNPT1	10	0.0085
DYNLL2	10	0.0077
DISC1	10	0.0072
SOX6	9	0.0092
ARIH1	9	0.0073
EXOC8	8	0.0088
TAZ	8	0.0088
GIGYF1	8	0.0084
FRK	8	0.0079

SPRED3	8	0.0071
TRIB1	8	0.0071

OSCC, oral squamous cell carcinoma.
